# The Impact of Pronator Quadratus Origin Release on the Clinical Outcomes of Scaphoid Nonunion Patients Treated with Pronator Quadratus Pedicled Bone Grafts

**DOI:** 10.3390/jcm13175157

**Published:** 2024-08-30

**Authors:** Ahmed Majid Heydar, Mustafa Kürklü

**Affiliations:** 1 Department of Orthopedic Surgery, Okayama Rosai Hospital, 1-10-25 Chikkomidorimachi, Minami Ward Okayama, Okayama 702-8055, Japan; 2 Orthopedic and Traumatology Clinic, Memorial Bahçelievler Hospital, Bahçelievler Merkez, Adnan Kahveci Blv. No: 227, 34180 İstanbul, Turkey

**Keywords:** scaphoid nonunion, vascularized bone graft, pedicled bone graft, pronator quadratus release

## Abstract

**Background/Objectives**: A pronator quadratus pedicled bone graft (PQPBG) is a distal radius volar vascularized bone graft used not only for avascular necrosis of the lunate but also for scaphoid nonunion. Despite its potency and its possession of a muscular shield, this vascularized graft has a disadvantage in that the potential shortness of the muscular leash may limit the distal transfer of the bone graft. Releasing of the pronator quadratus (PQ) ulnar origin was used to enhance the distal mobility of the graft. We aimed to investigate the effect of a PQ release on the surgical outcomes of scaphoid nonunions that were operated on with the PQPBG technique. **Methods**: Patients with scaphoid nonunion that were treated with PQPBG from 2009 to 2020 were reviewed. Patient demographic characteristics, surgical notes, physical examinations, and radiological evaluation data were collected. Wrist range of motion, grip strength, modified Mayo wrist score, and Quick-DASH score were used to evaluate the outcomes. The included patients were divided into two groups based on the origin release status of their PQ, i.e., with and without release. **Results**: This study included 37 patients, 17 of whom underwent a PQ release and 20 of whom did not. The failure rates for the two groups were one and four patients, respectively, and there was no significant difference between them (*p* = 0.11). The postoperative mean wrist extension in the patients with a PQ release was significantly greater than that in the patients without a release (43.5 ± 6.8 vs. 36.5 ± 7.7, *p* = 0.0038). Although wrist flexion, ulnar deviation, radial deviation, mean outcome assessment scores, and grip strength were greater in the patients with a PQ release than in those without, no statistically significant intergroup differences were observed (*p* > 0.05). **Conclusions**: The PQPBG technique is a viable option for achieving bony union in patients with scaphoid nonunion, but it results in the postoperative restriction of wrist extension. PQ release during a graft transfer may have a favorable effect on both bone union and clinical outcomes.

## 1. Introduction

Scaphoid fractures are common upper extremity injuries, with an incidence of 29 per 100,000 people, and young active males constitute the riskiest group for sustaining these fractures [1]. These fractures tend to be nonunion and a considerable number, as high as 10%, of these fractures fail to form a union despite sufficient immobilization [2] and appropriate surgical fixation [3]. Most of the scaphoid surface is enclosed by chondral tissue, and the predominant retrograde nature of the blood supply and the presence of local destabilizing forces are considered the most common causes of nonunion predisposition [2]. In practice, the interval between the occurrence of the fracture and the commencement of treatment, as well as the presence of aseptic necrosis, are the primary factors that affect the bony union [4].

Scaphoid nonunion generally presents as persistent wrist pain and stiffness; nevertheless, not all cases are symptomatic, but symptoms may increase after strenuous use of the wrist [5]. Both a delay in presentation, with a consequent delay in the initiation of management, and persistent pathological mobility at the fracture site result in progressive bone resorption and cyst formation, with a consequential and considerable amount of bone loss that necessitates structural bone grafting to correct scaphoid shortening, achieve osseous fusion, and avoid the progression of osteoarthritic changes. Two types of bone graft interposition have been utilized to fill scaphoid nonunion bone defects: vascularized and nonvascularized bone grafts. Although successful results were attained via the use of nonvascularized bone grafts, most of the failure cases were due to the avascular necrosis of the proximal fragment [6]. Vascularized bone grafts are usually recommended for avascular necrosis-associated scaphoid nonunion and for patients who fail the nonunion surgery [7].

The pronator quadratus pedicled bone graft (PQPBG) technique is a distal radius volar pedicled vascularized bone graft that was first defined by Braun and Chacha for the treatment of scaphoid nonunion and Kienböck’s disease [8,9]. The more potent pedicle with a muscular shield differentiates PQPBG from other volar pedicle vascularized bone grafts [10]. However, the most significant limitation of such a graft is the potential shortness of the muscular leash, which limits the distal rotation of the graft [11]. To address this limitation, Kawai and Yamamoto [8] modified the original technique and described the release of the pronator quadratus muscle, through the subperiosteal dissection of the muscle from the ulnar origin, to increase the distal mobility of the graft. The impact of this muscle release on the surgical outcome was not studied. Therefore, we aimed to investigate the effect of pronator quadratus origin release on the surgical outcomes of scaphoid nonunions treated with the PQPBG technique.

## 2. Materials and Methods

**Figure 1:**
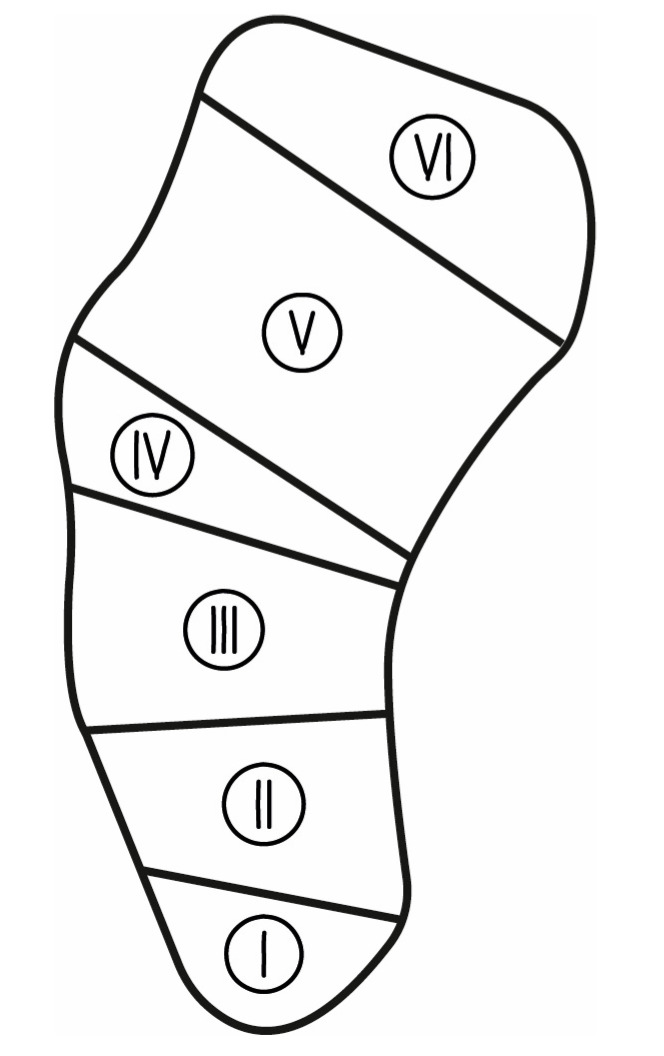
Schematic representation of the scaphoid anatomic locations used in the Schernberg classification are illustrated as follows: I—proximal pole, II—proximal waist, III—waist, IV—distal waist, V—distal pole, and VI—tubercle of the scaphoid.

Following the approval of this study by the local institutional ethics committee, a retrospective outcome study, using prospectively collected data from our institution and the senior author’s surgical database, was performed for patients with symptomatic scaphoid nonunion who had been treated with PQPBG between 2009 and 2020. All the patients were operated on and followed up on by the senior author (M.K.). In the scanned database, operation notes, clinical records, and radiologic images were used to assess the surgical technique, clinical outcome, and radiologic union of the scaphoid. The inclusion criteria for this study were symptomatic scaphoid nonunion with or without avascular necrosis, bone loss, hump-back deformity, or marked carpal instability, and they were treated with PQPBG. The presence of scaphoid nonunion for at least 6 months from the initial injury and a minimum postoperative follow-up of 10 months were necessary for inclusion. To maintain homogeneity, patients with advanced osteoarthritis or carpal collapse and those with previous wrist collapse were excluded. Interrupted follow-up or inaccessibility of medical records were also among the exclusion criteria. The cases of scaphoid nonunion were classified according to the nature and location of the nonunion; the Schernberg classification (Figure 1) [12] was used to determine the zone of the nonunion and the type of nonunion was classified according to Filan and Herbert [13] (Table 1).

**Figure 2:**
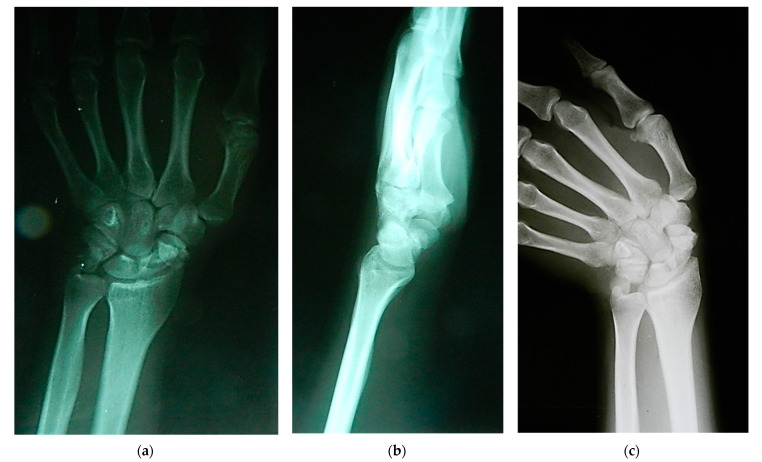
Radiographs showing scaphoid nonunion: (**a**) posteroanterior; (**b**) lateral; and (**c**) scaphoid radiographic views.

Scaphoid nonunion was defined as the absence of bony bridging the fracture for at least 6 months after the injury. The clinical diagnosis was confirmed by posteroanterior, lateral, and scaphoid radiographic views (Figure 2), whereas avascular necrosis of the scaphoid segment was diagnosed by contrast-enhanced MRI and confirmed intraoperatively by the lack of punctate bleeding at the fracture line. 

In the final follow-up, in addition to the patients being evaluated via objective parameters, including wrist range of motion in all directions and grip strength, both a physician-based scoring system and patient-reported outcome measures were used in the outcome assessment. The wrist range of motion was measured by a goniometer, and the grip strength was calculated by a JAMAR hand dynamometer. The modified Mayo wrist score (MMWS) [14] is a physician-based scoring system that has a total of 100 points equally distributed among four different assessments: pain, active flexion–extension range, and grip strength as a percentage of the normal side, as well as the ability to resume employment or normal daily activities. Higher scores signify better outcomes. The outcome was regarded as excellent when the score ranged from 90–100, whereas scores ranging from 80–89, 65–79, and below 65 points indicated good, fair, and poor outcomes, respectively. Similarly, the shortened disabilities of the arm, shoulder, and hand questionnaire (Quick-DASH) [15] was used as a patient-reported outcome measure to assess patients’ perceptions of their wrist function. It contains 11 questions evaluating present symptoms, sleep, difficulty in performing specific tasks, social activities, and work function. The score also ranges from 0 to 100 points, but unlike the MMWS, higher scores indicate worse wrist function. All the values obtained from the final follow-up assessment were compared with the corresponding preoperative findings and analyzed statistically.

For comparison purposes, all included patients were divided into 2 groups based on the preference of the surgeon, which was dictated by the length of pedicle measured during surgery: PQPBG with and without pronator quadratus ulnar origin release. The outcomes of these patients were compared.

### 2.1. Surgical Technique

Under general anesthesia, following baseline preparation and draping, the patient was placed in a supine surgical position with the supinated upper limb on a radiolucent operating arm table. The operation was performed under tourniquet control, and the arm was exsanguinated with sterile esmarch. A volar-angled skin incision, centered on the scaphoid tubercle and extending proximally over the flexor carpi ulnaris tendon, was made and the incision angled toward the base of the first metacarpal distal to the scaphoid tubercle. The radioscaphocapitate ligament complex was then split obliquely from the tubercle toward the radius rim and preserved for later repair. The scaphoid nonunion was identified, the fracture edges were refreshed, the sclerotic bone ends were removed with a bone curette, and the interposed fibrous soft tissue was debrided with a fine-nosed rongeur. Following sufficient debridement until the healthy cancellous bone was exposed, the tourniquet was deflated to assess the circulation of the proximal fragment. In the absence of punctate bleeding, the diagnosis of avascular necrosis was confirmed. The extent of the scaphoid bone defect was subsequently evaluated, and accordingly, the size of the pronator quadratus graft, which is usually approximately 15–20 mm long, was outlined at the radius styloid in the most radial and distal insertion of the pronator quadratus muscle. Kirschner wires were used to make holes at the borders of the outlined graft to facilitate bone harvesting via a fine osteotome without detachment of the muscle from the graft. The graft was mobilized by dissecting the pronator muscle toward the ulna parallel to its distal insertion, resulting in a 20 mm thick pedicle.

**Figure 3:**
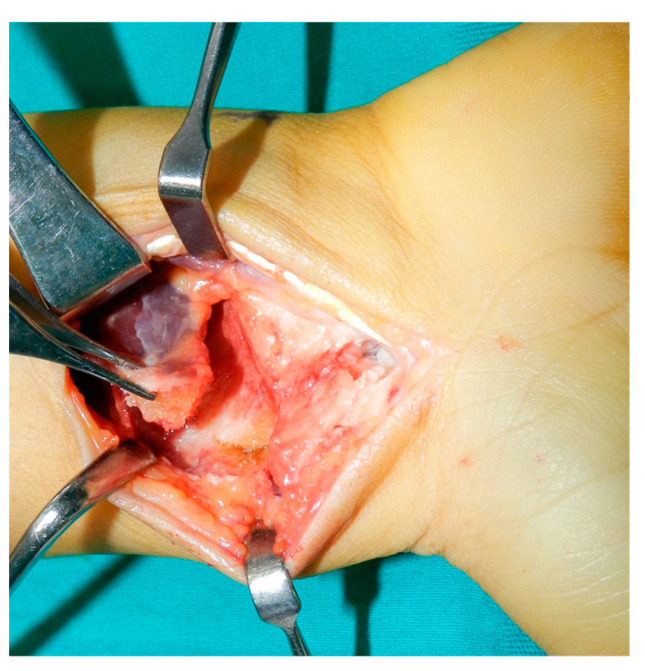
Intraoperative image showing short muscle pedicle that could not be grafted in the recipient site and skin marking for separate skin incision for releasing the muscle distal ulnar origin.

When the muscle pedicle was very short and tight to the recipient site for transfer (Figure 3), pronator quadratus muscle release was performed through a separate incision over the distal ulna and subperiosteal dissection of the ulnar origin of the muscle. Next, the scaphoid bone was reduced by applying traction force to the thumb, and direct manipulation was performed to correct and rotate the deformity. The graft was inserted into the scaphoid defect; if the size of the graft and the insertion pressure were adequate, partial stability was achieved once the applied longitudinal traction was released. Under fluoroscopic guidance, fixation was achieved either by two to three 1.2 mm k wires or a 3 mm headless compression screw introduced retrogradely from the scaphoid tuberosity and distal fragment. The graft was further secured by suturing its muscle to the radioscaphocapitate ligament complex. The ligament complex was then repaired, and the skin was closed.

### 2.2. Postoperative Care and Evaluation

**Figure 4:**
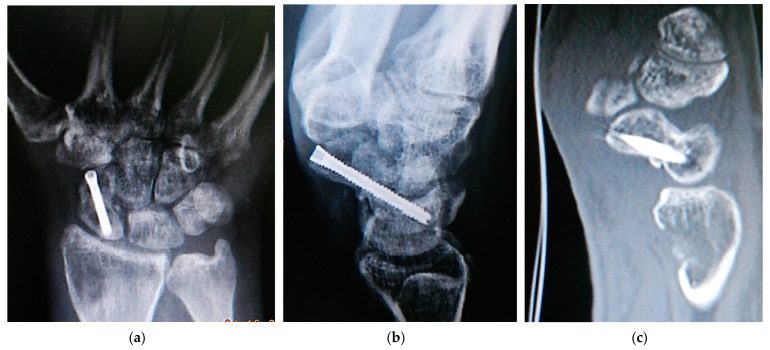
Postoperative radiologic investigation of scaphoid nonunion with resultant union achievement: (**a**) posteroanterior and (**b**) lateral x-rays showing scaphoid union and bone defect in the donor site of the distal Radius; (**c**) sagittal CT scan view showing incorporation of the pronator quadratus graft and the donor site bone defect.

Strict immobilization with a short-arm cast including the first metacarpopharyngeal joint was performed for 10–12 weeks. In cases where K wires were used for fixation, they were removed at the time of cast removal. Then, physical therapy for active range of motion and muscle strengthening was initiated, and the patients were allowed to use their wrists for daily activities, while heavy loading was still postponed until the sixth month after surgery, based on the surgeon’s clinical and radiologic assessment. Immediate postoperative radiographs were used to assess the overall surgical procedure, whereas radiologic evaluations of union and graft incorporation were performed at 1.5, 3, and 6 months postoperatively. The presence of bony trabeculae joining the graft with both scaphoid fragments across the pre-existing nonunion site (Figure 4) and the absence of anatomic snuffbox tenderness were used to confirm the achievement of scaphoid union. Postoperative CT scans and contrast-enhanced MR imaging were reserved for suspected nonunion after the 6th month of surgery.

### 2.3. Statistical Analysis

The Shapiro–Wilk test was used to assess the normality of continuous data, which are presented as the means ± standard deviations. To evaluate within-group differences between preoperative and postoperative measurements, i.e., improvements from baseline, a paired *t*-test was utilized. Moreover, between-group differences were evaluated by the Student’s *t*-test or the Mann–Whitney U test. For categorical data, between-group differences were evaluated via the chi-square test. All the statistical tests were two-sided, and a *p*-value equal to or less than 0.05 was considered significant. All the analyses were conducted via SPSS 1 13.0 (SPSS Inc., Chicago, IL, USA).

## 3. Results

**Figure 5:**
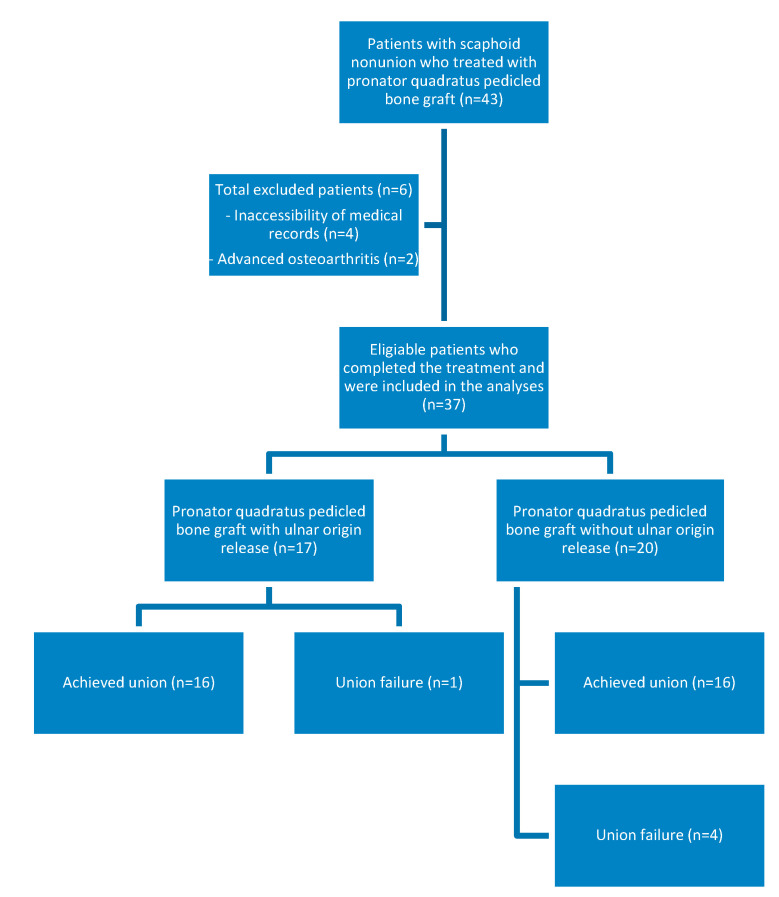
Flow diagram of this study.

The PQPBG graft technique was performed for 43 patients with scaphoid nonunion, but only 37 patients were eligible for study inclusion (inaccessibility of medical records for 4 patients and 2 patients who had advanced osteoarthritis) (Figure 5). Twenty-nine of the included patients were male, and eight were female. The mean age of the included patients was 24.4 ± 5 (20–39) years. The cause of the fracture was a fall on an outstretched hand in 17 patients, a traffic accident in 11 patients, and sport-related injury in 9 patients. The initial fracture was unrecognized in 12 patients and was treated with a short-arm cast in 25 patients. None of the patients had previously undergone surgical intervention for the initial fracture or nonunion. In total, 14 patients had scaphoid fractures located in the proximal waist (zone II), 16 in the waist (zone III), and 7 in the distal pole (zone V). The nonunion type was D1 (fibrous union) in 7 patients, D2 (pseudoarthrosis) in 7 patients, D3 (sclerotic nonunion) in 8 patients, and D4 (avascular necrosis) in 15 patients. The mean time from initial injury to surgery was 16 ± 5.7 (8–26) months and the mean follow-up period was 15.5 ± 5 (9.5–24) months.

Bony union was observed in 32 patients, and the average time to union was 4.5 ± 1 (3.9–6.5) months. Although flexion, radial, and ulnar deviations of the wrist improved from 51.3 ± 9.7°, 11.4 ± 4°, and 17 ± 5° to 58.2 ± 11.3° (*p* = 0.0036), 15.7 ± 6° (*p* = 0.0026), and 24.8 ± 6.6° (*p* < 0.0001), respectively, extension of the wrist significantly decreased from 52.5 ± 11.3° to 39.7 ± 8° (*p* < 0.0001) (Table 2). Three of the five patients who experienced union failure underwent four-corner arthrodesis, while the remaining two did not consent to the revision procedure and were lost to follow-up. During the course of treatment, no patient developed a postoperative infection, but only six patients developed complex regional pain syndrome and recovered completely conservatively.

The 37 patients were subdivided into two groups: 17 patients had a PQPBG with a pronator quadratus ulnar origin release (group 1), and 20 had a PQPBG without such a release (group 2). There was no statistically significant difference between the two groups in terms of age, sex, laterality, hand dominance, mechanism of fracture, nonunion type, or trauma to surgery time (*p* > 0.05). The baseline demographics and preoperative characteristics of both groups are shown in Table 3.

Postoperatively, one scaphoid in group 1 and four in group 2 failed to union, although the increased number of failures in group 2 was not significantly different between the two groups (*p* = 0.11). The mean degree of flexion, as well as the degree of radial and ulnar deviation, was greater in group 1 than in group 2, but the difference was not statistically significant (*p* > 0.05). In contrast, the mean extension arc was significantly lower in group 1 than in group 2. Additionally, the mean outcome assessment scores and grip strength were greater in group 1; however, no statistically significant intergroup differences were observed (*p* > 0.05). Comparisons of the fixation method, time to union, range of motion, outcome scores, and grip strength between the two groups and their differences in significance are shown in Table 4.

## 4. Discussion

In the present study, we investigated the union rate and clinical outcomes associated with using a pedicled bone graft from the pronator quadratus in the treatment of scaphoid nonunion. Additionally, we sought to determine whether releasing the pronator quadratus from its ulnar origin during the graft transfer had any effect on the outcomes achieved. Our results revealed that although a high union rate was achieved by such a vascularized bone graft, a significant wrist extension limitation was evident. This extension limitation was significantly greater in the patients who underwent a graft transfer without a pronator quadratus release. Furthermore, the patients who underwent pronator quadratus release demonstrated improved outcomes in terms of both bony unions and clinical results, although these findings were not statistically significant.

The substantial failure rate of scaphoid nonunion, which may reach 20% or even 38% in instances of avascular necrosis in patients receiving nonvascularized bone grafts and internal fixation [16], has led hand surgeons to rely on vascularized bone grafts to address these complex cases. This reliance is based on the viability of cortical osteocytes, increased bone strength, and elastic modulus [10], as well as the achievement of primary bone healing rather than creeping substitution and absorption, which are observed in nonvascularized bone grafts, resulting in quicker graft incorporation and bone union processes [17]. However, vascularized grafts do not consistently produce the desired outcome. According to a previous study, out of the ten patients treated with a dorsal carpal vascularized bone graft, only six achieved union [18]. Additionally, Straw et al. reported a union rate of only 27% in 22 patients treated with the same graft [19]. Therefore, several techniques for vascularized bone grafts have been described, but there is no consensus on which technique is best for the treatment of chronic scaphoid nonunion. They are broadly divided into free and pedicled vascularized bone grafts. Many surgeons prefer pedicled vascularized bone grafts because of the absence of donor-side morbidity, their reduced technical complexity, and the shorter operative time needed.

The advantages of volar pedicled bone grafts are the avoidance of blood vessels entering the dorsal ridge of the scaphoid, the ability to restore the scaphoid anatomy by correcting hump-back deformities [20], and the consistency of the anatomy of the volar carpal vessel [21]. The most commonly utilized volar pedicled bone grafts are the volar carpal artery and PQPBG. Compared with the volar carpal artery graft, the pronator quadratus graft offers the advantages of a sturdier pedicle, which has relatively small vessels and no muscle shield [10]. However, a considerable union failure rate of approximately 13.5% was reported in this study, whereas surprisingly low failure rates of 0–7% were reported in other published series [22,23,24]. This discrepancy in failure rates among the studies may be attributed to the fact that many factors, such as surgical technique, patient age, fracture displacement, presence of avascular necrosis, degree of instability, and smoking history, affect the union rate [24,25]. Although the statistical significance of the difference in radiologic union time is not apparent (*p* = 0.0548), the shorter union time observed in the patients without a pronator quadratus origin release is nearly significant. The reason for this shorter union time remains unclear, but it may be attributed to the fact that a greater number of the patients in this group underwent a rigid fixation with headless screws rather than a K wire. Although no studies directly compare the union of scaphoids when the pronator quadratus graft is fixed with headless screws and K wires, Lee et al. reported a shorter mean union time in their series when headless screws were used [22].

**Figure 6:**
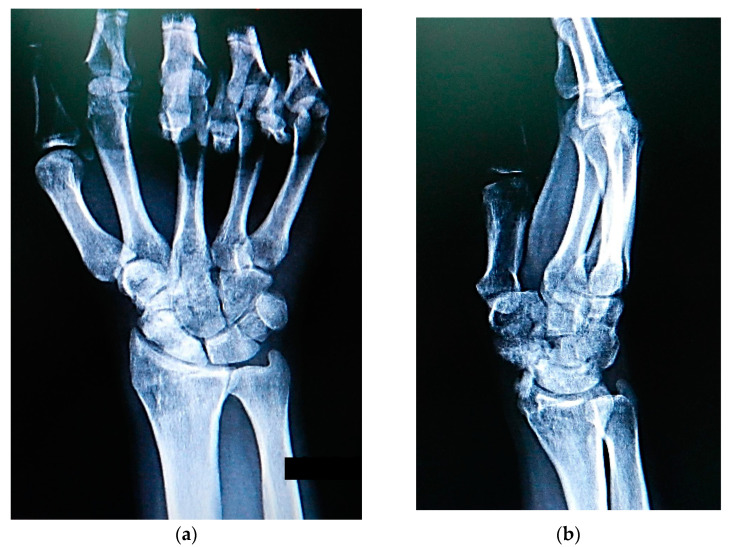
(**a**)Posteroanterior and (**b**) lateral X-rays revealing complete bony union of the scaphoid nonunion and calcification of the pronator quadratus muscle at 6th month postoperatively.

The most typical clinical sign of scaphoid nonunion is diminished wrist mobility [26], and one of the therapeutic goals is to increase the range of wrist motion. Research has suggested that an anterior approach for treating scaphoid nonunion results in the smallest loss of extension and flexion arcs [27]. However, other studies have shown that patients who undergo treatment with a PQPBG experience postoperative limitations in wrist motion, which are likely due to the muscle leash being sutured across the joint [28]. In our study, although flexion, ulnar, and radial deviations improved, we observed further postoperative limitations of restricted wrist extension, which contrasts with the findings reported by other studies [22,23,24] that reported postoperative improvements in all wrist movements, including extension. Limitations in one direction of motion and improvements in the other motions suggest that the limitation is not due to prolonged postoperative immobilization. We believe that the presence of the muscular leash crossing the joint volar and its possible shortening and stiffening over time, as revealed by radiologic calcification (Figure 6), may be the cause of this limitation.

Previous research has indicated that a minimum of 60° of wrist extension is necessary for individuals to perform activities of daily living [29]. Other studies have also demonstrated a direct relationship between a restricted wrist range of motion and a functional disability [30]. Furthermore, the perceived disability resulting from the restricted wrist motion has been found to be greater than the measured functional loss using common physical tests and outcome surveys [31]. It is important to counsel patients with scaphoid nonunion who are willing to undergo the PQPBG procedure about this potential loss, which may be particularly significant for athletes and heavy manual workers.

One of the notable limitations of the PQPBG technique is the shortness of the pedicles, which can restrict graft mobility and make it challenging to transfer the graft to the recipient site without creating excessive tension. Many researchers have attempted to address this issue by removing the pronator quadratus invested fascia, palmarly flexing the wrist, and pronating the forearm, or creating a shallow groove in the distal radius to host the pedicle [32]. However, subperiosteal dissection of the pronator quadratus distal ulnar origin, as described by Kawa and Yamamoto [8], is considered the most effective method for increasing pedicle mobility. While the effect of such a release on outcomes has not been studied extensively, careful literature screening revealed that various outcome-reporting series mentioned that when the muscle was too tight to transfer, the ulnar origin of the muscle should be subperiosteally released without providing any further information [22,23,24]. In the present study, we demonstrated that the patients who underwent a pronator quadratus origin release had less wrist extension loss than those who did not release the pronator quadratus. Additionally, pronator quadratus pedicle grafts with original release may yield better outcome assessment scores, grip strength, and range of wrist motion. On the other hand, grafts without release may be associated with a higher rate of union failure, which may be attributed to the tight pedicle compromising graft circulation.

One of the strengths of this study is that it highlights an option in a technique that has not been extensively researched and explores its possible impact on the overall outcome. Despite this, this study is not without limitations. The retrospective study design, and the single-surgeon series, may be vulnerable to observer bias due to the absence of a control group. Also, utilizing data from the same institution potentially increases the probability of selection bias. The fixation method was not standardized, and two types of fixations were employed: K wire and headless compression screws, which may have affected the results. However, successful fixation and perfect outcomes have been reported for both methods [22,23]. The small sample size is considered the most significant limitation of this study, despite it being the second-largest sample size in the literature. It is challenging to collect data on a vast number of patients treated with a PQPBG because of the controversial optimal treatment for scaphoid nonunion, which involves various techniques and different grafts. Therefore, future studies with larger sample sizes and randomized prospective clinical studies investigating the impact of a pronator quadratus origin release on the outcome are warranted.

## 5. Conclusions

In conclusion, despite its considerable rate of union failure, which may reach 13.5%, and the resulting postoperative restriction of wrist extension, the PQPBG technique is a viable option for achieving bony union in patients with scaphoid nonunion. It has shown high levels of satisfaction, improved grip strength, and minimal complications. In addition, our results showed that the release of the ulnar origin of the pronator quadratus during the graft transfer may have a favorable effect on bone union and clinical outcomes.

## Figures and Tables

**Table 1:** Filan and Herbert’s classification of scaphoid nonunion.

Type of the Nonunion	Definition	Description
D1	Fibrous union	No deformity
D2	Pseudarthrosis	Early deformity
D3	Sclerotic pseudarthrosis	Advanced deformity
D4	Avascular necrosis	Fragmented proximal pole

**Table 2:** Comparison between preoperative and postoperative values of all patients treated with PQPBG.

	Range of Motion						
	Flexion	Extension	Radial Deviation	Ulnar Deviation	Grip Strength	MMWS	Quick-DASH
Preoperative values	51.3 ± 9.7°	52.5 ± 11.3°	11.4 ± 4°	17 ± 5°	14.9 ± 3.3	53.8 ± 15.5	44.8 ± 11
Postoperative values	58.2 ± 11.3°	39.7 ± 8°	15.7 ± 6°	24.8 ± 6.6°	28.3 ± 8.4	77.4 ± 18.7	14.6 ± 13.5
*p*-value	0.0036 *	<0.0001 *	0.0026 *	<0.0001 *	<0.0001 *	<0.0001 *	<0.0001 *

* *p* < 0.05 represents statistical significance, MMWS—modified Mayo wrist score, Quick-DASH—shortened disabilities of the arm, shoulder, and hand questionnaire.

**Table 3:** Comparison of baseline demographic and preoperative characteristics between the two groups.

Demographics and Preoperative Characteristics	Group 1 (n = 17)	Group 2 (n = 20)	*p*-Value
Patient, n/total	17/37	20/37	
Age, mean (SD), year	24.9 ± 5	26 ± 4.5	0.29
Male sex, n	14 (82%)	15 (75%)	0.3
Right laterality, n	11 (65%)	13 (65%)	0.49
Mechanism of fracture			
Fall on outstretched hand	8 (47%)	11 (55%)	0.45
Traffic accident	6 (35%)	5 (25%)	0.48
Sport-related injury	3 (18%)	4 (20%)	0.46
Nonunion location			
Proximal waist (zone II)	8 (47%)	6 (30%)	0.149
Distal waist (zone IV)	6 (35%)	10 (50%)	0.19
Distal pole (zone V)	3 (18%)	4 (20%)	0.43
Nonunion Type			
D1	3 (18%)	2 (10%)	0.43
D2	3 (18%)	6 (30%)	0.43
D3	5 (29%)	3 (15%)	0.15
D4	6 (35%)	9 (45%)	0.28
Trauma to surgery time, month	15.1 ± 5	16.7 ± 6.2	0.2161
Range of Motion, degree			
Flexion	51.5 ± 8.2	51.2 ± 10.9	0.4736
Extension	52 ± 9.8	53 ± 12.4	0.4037
Ulnar deviation	10.9 ± 3.9	11.7 ± 4.2	0.2689
Radial deviation	16.8 ± 4.8	17.2 ± 5.3	0.3908
Grip Strength, Kg	14.1 ± 3.2	15.5 ± 3.2	0.1081
MMWS, point	53.2 ± 17.5	54.3 ± 13.6	0.4241
Quick-DASH, point	43.3 ± 11.6	46.1 ± 10.2	0.2270

MMWS—modified Mayo wrist score, Quick-DASH—shortened disabilities of the arm, shoulder, and hand questionnaire.

**Table 4:** Comparison of postoperative outcomes between the two groups.

Postoperative Characteristics	Group 1	Group 2	*p*-Value
Screw fixation, patient n	3 (18%)	6 (30%)	0.1984
Follow up, month	16.8 ± 4.5	13.3 ± 5.4	0.0785
Radiologic union time	4.8 ± 0.8	4.2 ± 1	0.0548
Failure to union, n	1 (6%)	4 (20%)	0.1108
Range of Motion, degree			
Flexion	60.6 ± 10	56.2 ± 12	0.1292
Extension	43.5 ± 6.8	36.5 ± 7.7	0.0038 *
Radial deviation	13.2 ± 4.1	15.7 ± 6	0.0824
Ulnar deviation	23.5 ± 4.7	26 ± 7.7	0.1354
Grip Strength, Kg	29 ± 8.14	27.5 ± 8.5	0.2990
MMWS, point	81.1 ± 14.9	74.2 ± 20.8	0.1369
Excellent	5 (29%)	3 (15%)	
Good	6 (35%)	8 (40%)	
Fair	4 (24%)	5 (25%)	
Poor	2 (12%)	4 (20%)	
Quick-DASH, point	12.4 ± 9.7	16.4 ± 15.8	0.1896

* *p* < 0.05 represents statistical significance, MMWS—modified Mayo wrist score, Quick-DASH—shortened disabilities of the arm, shoulder, and hand questionnaire.

## Data Availability

The data presented in this study are available on request from the corresponding author due to patient privacy.
